# Impaired ILK Function Is Associated with Deficits in Hippocampal Based Memory and Synaptic Plasticity in a FASD Rat Model

**DOI:** 10.1371/journal.pone.0135700

**Published:** 2015-08-25

**Authors:** D. Bhattacharya, E. P. Dunaway, S. Bhattacharya, J. Bloemer, M. Buabeid, M. Escobar, V. Suppiramaniam, M. Dhanasekaran

**Affiliations:** 1 Department of Drug, Discovery and Development, Auburn University, Auburn, Alabama, United States of America; 2 Department of Psychology, Auburn University, Auburn, Alabama, United States of America; Georgia Regents University, UNITED STATES

## Abstract

Fetal Alcohol Spectrum Disorder (FASD) is an umbrella term that encompasses a wide range of anatomical and behavioral problems in children who are exposed to alcohol during the prenatal period. There is no effective treatment for FASD, because of lack of complete characterization of the cellular and molecular mechanisms underlying this condition. Alcohol has been previously characterized to affect integrins and growth factor signaling receptors. Integrin Linked Kinase (ILK) is an effector of integrin and growth-factor signaling which regulates various signaling processes. In FASD, a downstream effector of ILK, Glycogen Synthase Kinase 3β (GSK3β) remains highly active (reduced Ser^9^ phosphorylation). GSK3β has been known to modulate glutamate receptor trafficking and channel properties. Therefore, we hypothesize that the cognitive deficits accompanying FASD are associated with impairments in the ILK signaling pathway. Pregnant Sprague Dawley rats consumed a “moderate” amount of alcohol throughout gestation, or a calorie-equivalent sucrose solution. Contextual fear conditioning was used to evaluate memory performance in 32–33-day-old pups. Synaptic plasticity was assessed in the Schaffer Collateral pathway, and hippocampal protein lysates were used to evaluate ILK signaling. Alcohol exposed pups showed impaired contextual fear conditioning, as compared to control pups. This reduced memory performance was consistent with decrease in LTP as compared to controls. Hippocampal ILK activity and GSK3β Ser^21/9^ phosphorylation were significantly lower in alcohol-exposed pups than controls. Increased synaptic expression of GluR2 AMPA receptors was observed with immunoprecipitation of post-synaptic density protein 95 (PSD95). Furthermore, immunoprecipitation of ILK revealed a decreased interaction with GluR2. The ILK pathway appears to play a significant role in memory and synaptic plasticity impairments in FASD rats. These impairments appear to be mediated by reduced GSK3β regulation and increased synaptic stabilization of the calcium-impermeable GluR2 AMPA receptors.

## Introduction

Alcohol is probably the most commonly used and socially accepted psychoactive substance. However, alcohol consumption is not recommended during any stage of pregnancy; indeed, alcohol use during pregnancy can lead to a range of cognitive and physical consequences in the developing fetus [1]. Indeed, FASD are the leading cause of mental retardation in the United States. According to the United States Centers for Disease Control and Prevention, the prevalence of FASD in the U.S is relatively high (1.5–2.0 cases/1000 births), and exceeds that of other countries around the world [2].

Up to 94% of FASD children report mental health problems, and 79% have difficulties maintaining employment. The lifetime cost of each individual with FASD is estimated at $2 million [3]. Despite this great cost and the fact that the neuroanatomical and neurochemical effects of chronic alcoholism have been well elucidated, to date there are no therapeutic interventions available to treat FASD-induced cognitive deficits. FASD is fully preventable by abstaining from drinking during pregnancy; however, 100% compliance with this preventative measure may be difficult considering that the majority of FASD cases are the result of drinking prior to detecting the pregnancy. Furthermore, an estimated 9% of women will continue to drink despite the fact they know they are pregnant, some of them heavily (up to 0.3%) or in binges (up to 3%) [4]. Of great concern are “moderate” drinkers (women consuming a maximum of 7 drinks per week) since there is some debate as to whether moderate alcohol consumption is safe during pregnancy [5]. Hence, it is of extreme relevance to determine the mechanisms underlying FASD to develop optimal therapeutic interventions to address and potentially reverse the deleterious effects of alcohol exposure during gestation.

The two major cognitive consequences of FASD are reduced intelligence and memory impairments [6, 7]. FASD children are also at an increased risk of developing attention deficit hyperactive disorder (ADHD) and autism spectrum disorders (ASD), both of which are associated to significant learning disabilities and memory impairments [8]. At the root of these impairments may be the profound detrimental effect that prenatal alcohol exposure can have on the development of various brain regions associated with short- and long-term memory storage. Indeed, FASD can result in anatomical, biochemical and electrophysiological changes in the regions of the brain involved in memory formation and storage, namely the hippocampus and cortex [9].

Prenatal alcohol exposure also disrupts development of a variety of cellular processes, including insulin resistance and reduced neurotrophic factor expression. Insulin signaling is linked to ILK signaling through Phosphatidylinositol-4,5-bisphosphate 3-kinase (PI3K), downstream Protein Kinase B (Akt), and GSK3β phosphorylation [10]. Prenatal alcohol increases expression of the Phosphatase and tensin homolog (PTEN), a negative regulator of the PI3K pathway [11]. PTEN endogenously suppresses ILK activity, which is a downstream effector of integrins and insulin signaling. However, the role of ILK in prenatal alcohol-related deficits has not been investigated to date.

ILK appears to be localized with β1 integrins in humans and rodents [12]. ILK facilitates various cellular functions such as survival, cytoskeletal dynamics, and proliferation; thus, it has played a vital role in cancer research [13]. ILK activity is required to promote neurite growth factor (NGF) mediated neurite growth in rat pheochromocytoma cells; indeed, inhibition of ILK abolishes NGF-mediated neurite growth. Thus, ILK appears to play an important role in neurogenesis [14]. ILK appears to be involved in cocaine-induced synaptic plasticity, possibly via an interaction with subunits of the α-Amino-3-hydroxy-5-methyl-4-isoxazolepropionic acid glutamate receptors (AMPAR) and PSD95 proteins. Such interaction proves crucial for addiction-related modulation of synaptic plasticity and memory [15]. In neurodegenerative diseases (e.g., Alzheimer’s disease and Parkinson’s disease) characterized by learning and memory deficits, ILK-related mechanisms are compromised [16, 17]. Inhibitors of ILK have been found to increase Tau protein hyperphosphorylation (a hallmark of neurodegenerative disorders) through activation of GSK3β [14], highlighting the important role of ILK in the etiology of neurodegenerative disease. Despite the apparent relevance of ILK in conditions associated to memory deficits, its actual role in memory formation and synaptic plasticity has not been well elucidated. However, a few studies have provided evidence for a strong role of ILK function in learning and memory. For example, we have previously reported that ILK signaling through GSK3β is impaired in a diabetic model of insulin-resistant brain, with accompanying impairments in memory and synaptic plasticity [18].

The observations described above suggest that the ILK pathway could be the focus of a novel therapeutic target in FASD. Indeed, synaptic plasticity and cognitive function can be enhanced through different manipulations of ILK activity. For example, in aged rats, PI3K-ILK signaling can be potentiated with Brain derived neurotropic factor (BDNF) treatment to obtain enhanced synaptic plasticity and cognitive function [19], and ILK activation inhibits GSK3β activity and helps restore synaptic plasticity [19].

In the present study, we hypothesized that cognitive deficits accompanying moderate prenatal alcohol exposure is associated with impairments of the ILK pathway. We considered a continuous exposure of low level alcohol dose in rats as an appropriate model to study FAS like memory impairments. This model can indeed produce functional deficits in learning and memory [20]. The functional effects of this modulation were assessed using a contextual fear conditioning preparation, in which an environment is associated to the delivery of an electrical stimulus. Memory of the conditioning episode was correlated to ILK-GSK3β signaling and synaptic plasticity.

## Materials and Methods

### Subjects

The animal protocol and experiments were pre-approved by Auburn University IACUC committee (PRN# 2013–2265). We strictly adhered to the guidelines and directions mentioned in the protocol. Eight time-pregnant albino rats (200–250 g) (Sprague Dawley strain, Harlan Laboratories) consumed 10% (v/v) alcohol prepared with 95% ethyl alcohol and tap water throughout the gestation period, starting from the second day of gestation. The alcohol solution was sweetened with 3% glucose and 0.125% saccharin (Sigma Aldrich, USA) [21]. Six Non-exposed time pregnant rats received an equivalent solution lacking the ethyl alcohol. Animals were housed in a vivarium maintained on a 12 h:12 h light:dark cycle (lights on at 6:00 am) and at a temperature of 22–24°C. Bottles filled with the alcohol solution were offered at 6:00 pm and fluid consumption was measured after 15 hours of free access to the solution (access to food was *ad lib*). Differences in liquid volume were converted to volume intakes after accounting for the ethanol solution density (weight in grams/0.9868). There were no differences in consumption or weight gain between dams receiving alcohol and sweetened water. This volume of alcohol is considered to be animal model of moderate alcohol consumption: The dams consumed around 20ml of ethanol solution (equivalent to 6g/kg/day) which is comparable to 1 to 2 drinks per day for an adult human [22]. The day after parturition was considered postnatal Day 1 (PND 1) and all litters were culled to ten pups per dam.

### Contextual fear conditioning

**Fig 1:**
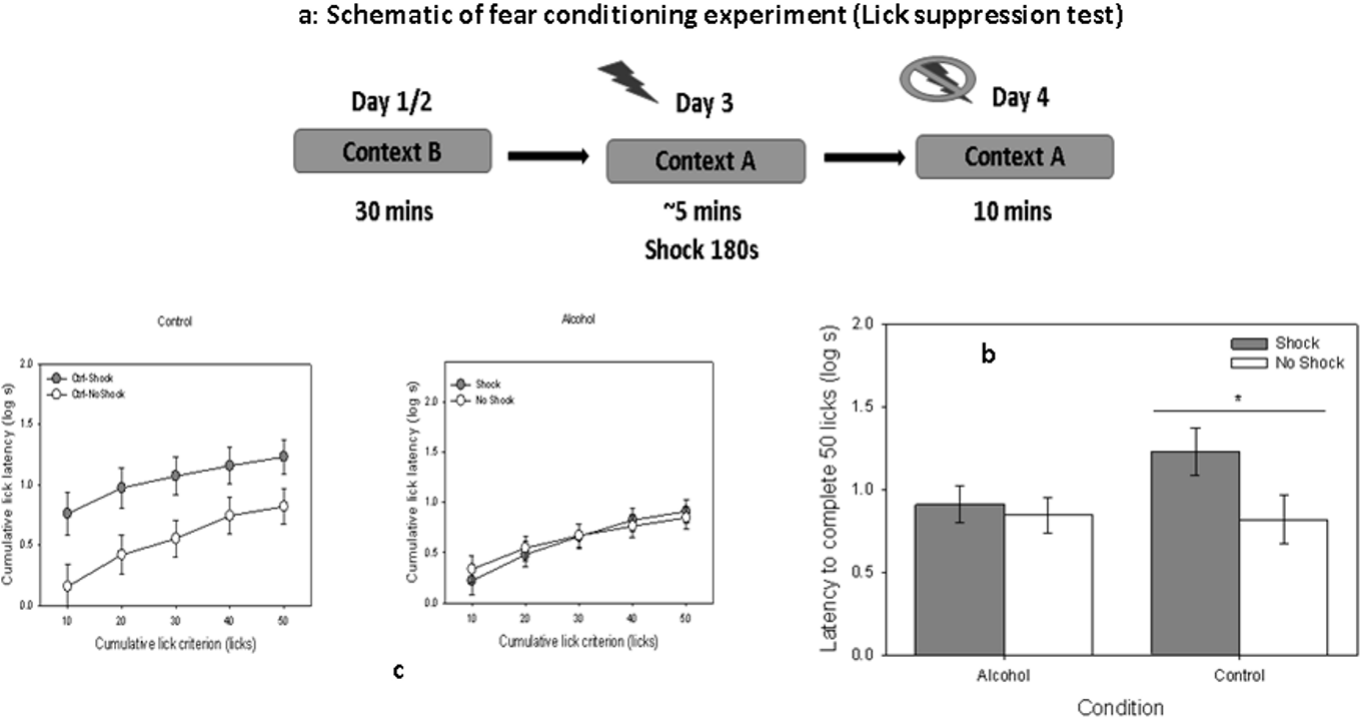
Prenatal alcohol impairs hippocampal-based contextual fear memory. (a) four day lick suppression behavioral assay on control (n = 10) and alcohol groups (n = 16). Days 1 and 2 constituted training of the licking response in Context B, followed by conditioning in Context A on Day 3. A 0.65-mA scrambled footshock was delivered after 180^th^ s of context exposure during the conditioning day. The latency to complete 50 licks in Context A were used as a measure of memory of the conditioning experience. (b) Time to complete 50 licks was calculated for the shock and no shock alcohol groups, compared to their nonexposed counterparts. (c) cumulative latencies to complete 50 licks are shown in 10 lick intervals for control and alcohol groups for shock and no shock category respectively. Differences were taken as statistically significant if *p* < 0.05.

Contextual fear conditioning is mainly dependent on hippocampal function. Dorsal hippocampus has a significant role in conditioned contextual freezing [23, 24]. Twenty-six animals served as subjects in this experiment. They were assigned to two groups, Alcohol (Eth, *n* = 16) or Control (Ctrl, *n* = 10). All animals were approximately 33 days old at the initiation of the behavioral portion of the study (average weight was 200 g). Each group was further randomly divided into shock and no shock subgroups. Animals were first trained to lick from a water spout in a novel environment, Context B. Conditioning was conducted in a different context, Context A, which was paired with a shock (Fig 1A). Memory of the association between Context A and the shock was assessed by placing the animals back in Context A, allowing them to begin licking from the spout, and recording the number of licks. The testing session was terminated at 10 min; thus, 600 s represent ceiling latency. Subjects’ access to water was gradually restricted to one hour per day over the week prior to initiation of the study, provided approximately one hour after completion of each day’s session. All conditioning procedures occurred in standard MedAssociates rat operant chambers and the experiments were scored by individuals who were blinded to the condition. Two modifications of these chambers served to create Context A (chambers with no additional cues), and Context B (a different set of chambers, modified using a striped pattern to cover the otherwise clear walls). Behavioral training and assessment occurred over 4 days, as follows. On Days 1 and 2 (lick training), subjects were trained to lick for water in Context B during two 30-min sessions. On Day 3 (conditioning), subjects were placed in Context A for 270 s. A 0.65-mA electric foot shock was delivered at 180 s for a duration of 2 s. Water bottles were not available during the conditioning session. On Day 4 (memory assessment), subjects were exposed to Context A for a 10 min period with water bottles available; this test evaluated recall of the association between Context A and the foot shock. Timing at which each lick was produced was recorded. Rats ‘freeze’ in anticipation of shock; thus, longer latencies to drink were assumed to reflect higher expectation of shock to be delivered in Context A (i.e., stronger memory of the conditioning experience). Latencies are skewed to the right; thus, a log (base 10) transformation was applied to the data to meet the normality criterion of parametric statistics. A 2 (treatment: alcohol vs. control) x 2 (condition: shock vs. no shock) analysis of variance (ANOVA) was conducted on the latency to complete 50 licks, assessed from the moment the first lick was produced until the 50th lick was produced.

### Electrophysiology recording

Rats were euthanized by decapitation and the brains rapidly removed and placed in ice-cold cutting solution containing (in mM) 120 NaCl, 11 D-Glucose, 26 NaHCO_3_, 6 MgCl_2_, 3 KCl, 0.5 CaCl_2_, 5 HEPES and 0.3 kynurenic acid. Coronal slices (300 mM) were cut with a Leica VT-1200S in the cutting solution. The slices were then transferred to artificial cerebrospinal fluid (aCSF, in mM 124 NaCl, 3 KCl, 1.5 MgSO_4_7H_2_0, 1.2 NaH_2_PO_4_, 2.4 CaCl_2_, 26 NaHCO_3_, and 10 D-Glucose bubbled with 95%CO_2_/5%O_2_) for at least 1 h and then maintained at room temperature until use.

### Measurement of Basal Synaptic Transmission and Long Term Potentiation

Following incubation, electrophysiological recordings were performed in a submerged recording chamber with continuous perfusion with aCSF (2–3ml/min) bubbled with 95%CO_2_/5%O_2_ carbogen, maintained at room temperature. fEPSPs were recorded from Schaffer collateral pathway SC-CA1 synapses with a glass pipette filled with aCSF (2–4MΩ). Stimulating pulses were applied at Schaffer collaterals via a bipolar stimulating electrode positioned 300 μm closer to CA3 subfield than the recording electrode. After placing stimulating and recording electrodes in the CA3 and CA1 regions respectively, stimulus intensity was lowered to the point where the fEPSP disappeared completely leaving the stimulus artifact intact. For stimulus response curves, current intensity was altered from 0–120μA. For LTP experiments, baseline was recorded at 50% of amplitude at which the initial population spike appeared. LTP was induced after 15 mins of stable baseline recording using a Theta Burst Stimulation protocol (TBS), and recording was continued for 60 mins post TBS. All electrophysiological data are presented as means ± SEM. For plasticity experiments, significance was determined using one-way ANOVA, followed by comparisons using a Bonferroni correction.

### Immunoprecipitation (IP) assay

PSD95 was immunoprecipitated from pooled hippocampal tissue lysate using 1:10 anti-PSD95 (Santa-Cruz) antibody coated on Pure-Proteome A/G magnetic beads (Millipore), and using vendor-supplied direct IP protocol. The immunoprecipitated fraction was purified through several washing steps with 1X IMP buffer, pH = 7.4. Finally, beads were boiled in 50ul of Laemmle Buffer at 70°C and separated on SDS PAGE, which was followed by western blot analysis.

### Western Blot analysis

The PSD95 immunoprecipitation assay was probed with PSD-95 rabbit primary antibody (1:1000, Cell Signaling) to check for presence of PSD-95 pulled down. Equal amounts of sample were loaded to probe interaction of PSD-95 with GluR2 (Millipore), GluR1, and ILK with rabbit primary antibodies (1:1000, Cell Signaling). Whole hippocampal protein lysates were probed for BDNF or proBDNF, and GSK3β to total GSK3β, using their respective primary antibodies at 1:1000 (Cell Signaling). All blots were probed with Dy-Light 660 anti-rabbit secondary antibody (1:10000, Thermo Scientific) using a Fuji FLA 5100 scanner. They are presented as means ± SEM. Significance was determined using a two-tailed Student's t-test.

### ILK activity assay

ILK activity was determined in hippocampal tissue homogenates using an immune complex kinase assay [21–24]. Briefly, tissue lysates were pooled and incubated with 1:50 anti-ILK mAb (cell signaling). The resulting immune complexes were washed three times in kinase reaction buffer, followed by incubation with 1 μg inactive Akt and ATP (final concentration: 200 μM) in 50ul kinase reaction buffer for 1 h at 30°C. The reaction products (supernatant) were resolved on SDS/PAGE. The beads were then processed as described in IP assay for ILK immunoblot. Membranes were probed with antiphospho-Akt (ser^473^) mAb (Cell Signaling Technology). The blot was developed using Dy-Light 660 anti-rabbit secondary antibody (1:10000, Thermo Scientific) using a Fuji FLA 5100 scanner. The data are presented as means ± SEM. Significance was determined using a two-tailed Student's t-test.

## Results

### Effect of prenatal alcohol on litter size and body weight

There was no difference in the number of pups per litter (approximately 4–5 male pups per dam) and body weight observed between the prenatal alcohol exposed and nonexposed rats.

### Moderate prenatal exposure to alcohol resulted in impairments in hippocampal-based contextual fear memory

Previous studies on the behavioral outcomes of prenatal exposure to alcohol suggest that moderate drinking impairs spatial memory [25–27]. However, more recent studies suggest such impairments occur even after moderate to low dose prenatal exposure [26]. This controversy is not easily addressed because these reports either included different cognitive tasks or the age of the models used was different; thus, further studies are warranted. In the present study we assessed the effects of prenatal exposure to ethanol on memory, using a contextual fear conditioning preparation, assessed as disruption of licking behavior. The effects of treatment (alcohol *vs*. control) and condition (shock *vs*. no shock) did not reach statistical significance, *F*s_1,51_ = 1/74 and 3.16, *p*s = 0.19 and 0.08, respectively. However, the interaction of these two factors was significant, *F*_1,51_ = 6.85, p < 0.05 (Fig 1B). Thus, level of fear observed in the shock vs. no shock animals was dependent on whether they had been exposed to alcohol during the prenatal period. This conclusion was confirmed with pairwise planned comparisons, which revealed that the shock and no shock conditions differed in the control, *F*_1, 51_ = 7.59, p < .01, but not the alcohol condition, *F*_1, 51_ < 1 (Fig 1C).

### Moderate prenatal alcohol impaired synaptic plasticity in the hippocampus

**Fig 2:**
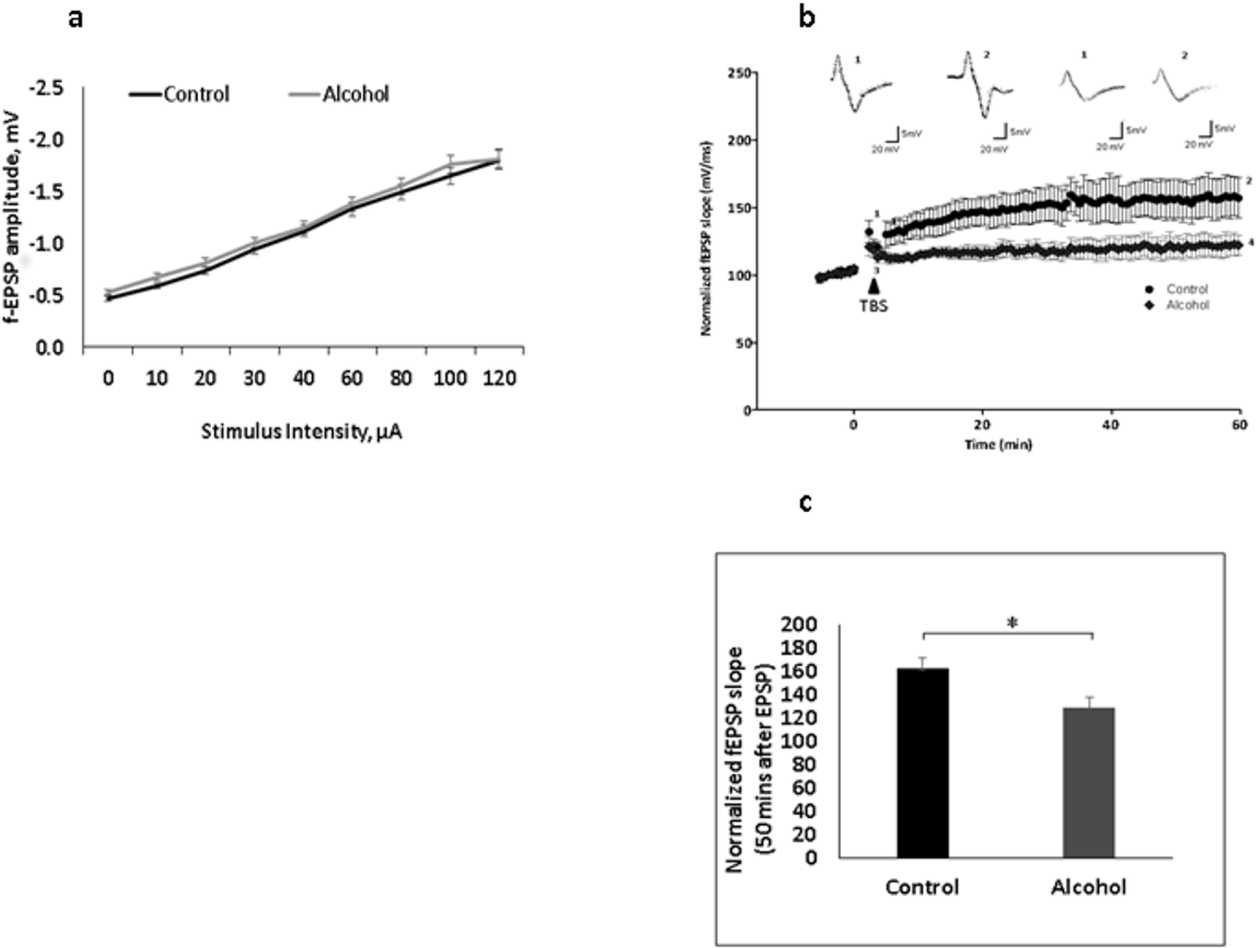
Prenatal alcohol impairs synaptic plasticity. (a) Input-output curves showing hippocampal basal synaptic transmission did not change in rats prenatally exposed to alcohol compared to controls. The graph shows f-EPSP amplitudes (mean ± SEM) as a function of stimulus intensity in the CA1 stratum radiatum. (b) TBS-induced LTP was recorded in hippocampal brain slices from prenatal alcohol exposed and nonexposed control rats (*n = 5*). The figure presents the time-course of percentage change in field EPSP slopes (mv/ms) with representative traces. The arrow indicates the time at which TBS protocol was delivered and 5 minute baseline is shown for clarity. Inset, representative fEPSP traces taken before the TBS (1,3) and after stabilization of LTP expression (2,4) for control and alcohol groups accordingly. (c) LTP was reduced in animals exposed to prenatal alcohol as compared to the nonexposed controls; [average for exposed animals was 162.2 ± 19.0%, and for nonexposed animals was 129.6 ± 20%, *F* = 6.217; *p* < 0.05].

Deficits in hippocampus-dependent associative learning and memory have been supported with long-term potentiation (LTP) in the Schaffer collateral pathway. We measured the changes in field potential in one-month old rat brains. In animals exposed to alcohol during the prenatal period, both moderate and binge drinking have been shown to result in deficits in cognitive function and neurogenesis [28, 29]. However, some studies questioned the amount of alcohol intake that can influence the deficits in memory impairment [30–32]. The differences in the pattern of administration of alcohol could also be a reason for the differences seen in behavioral studies. In this study, we looked into moderate continuous drinking, in which the animal has access to an alcohol solution as their only source of fluid. Following this administration regime, we initially determined the overall CA3-CA1 synaptic neurotransmission in the brain of animals prenatally exposed to alcohol, as well as in control rats. We monitored input-output (I/O) curves as a measure of overall basal synaptic transmission. Prenatal exposure to alcohol had no impact on I/O (*p* = .96; Fig 2A; also see [33]), which appears to be affected in acute or binge alcohol consumption models [34]. LTP was recorded from the Schaffer collateral pyramidal cell region at CA1 using theta burst stimulation (TBS) applied at CA3. Moderate prenatal alcohol decreased LTP measured as percentage change of fEPSP from baseline as compared to the unexposed controls (Fig 2B and 2C; 121.453 ± 8.2% vs 167.188± 16.5%, *p* < 0.05). LTP induction was lowered in exposed animals, which suggests a possible modulation of glutamate receptors at the synapse.

### Moderate Prenatal alcohol exposure reduced ILK activity but not expression

**Fig 3:**
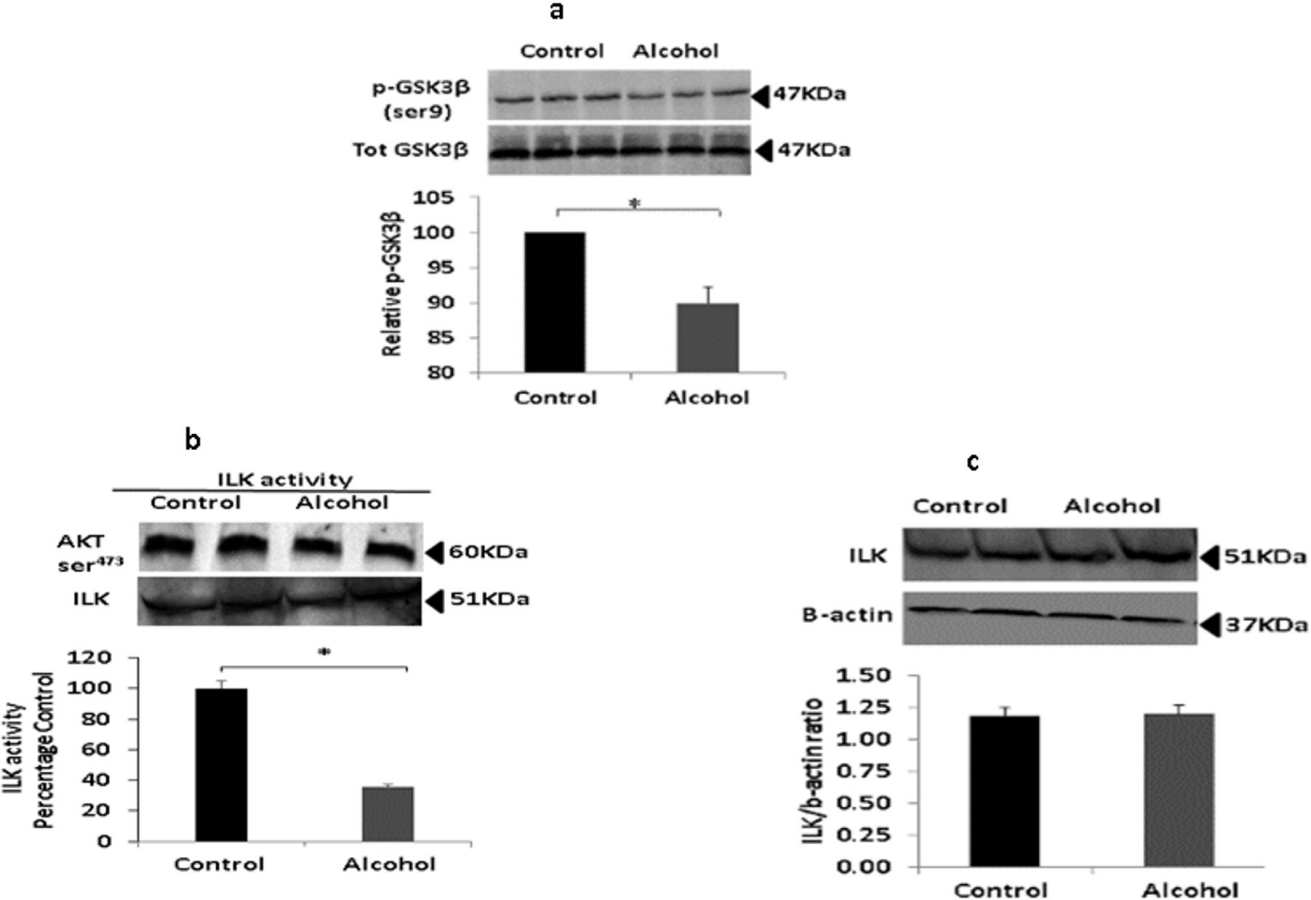
Prenatal alcohol impairs ILK activity. (a) Western blot analysis of total GSK3β and its Ser^9^ phosphorylation state was performed in hippocampal lysates from rats prenatally exposed to alcohol and nonexposed controls (*n* = 4). There was a significant decrease in the pGSK3β/GSK3β ratio in exposed rats, suggesting increased GSK3β activity as a result of alcohol exposure (*p* < 0.05). (b) Western blot analysis of expression of ILK in brain hippocampal protein lysates from exposed and nonexposed rats (*n* = 4). There were no differences in densitometric evaluation shows in expression of ILK as a result of alcohol exposure. (c) ILK activity assay was performed with pooled hippocampal protein lysates from control and alcohol rats (*n* = 4) and Akt ser^473^ phosphorylation assessed with western blot analysis. The quantitation of band density analysis shows reduced ILK activity in alcohol-exposed animals (*p* < 0.05).

Prenatal alcohol increases GSK3β activation [35], and GSK3β activity impairs learning and memory by modulating expression of surface receptor proteins (as well as affecting other physiological neuronal functions [36]). ILK phosphorylates both GSK3β and Akt (which is, in turn, the primary regulator of GSK3β phosphorylation) as downstream signaling molecules. Phosphorylation of GSK3β inhibits its activity [13]; thus, deficits in ILK should result in enhanced GSK3β and, consequently, impairments in learning and memory. However, the role of ILK in learning and memory after prenatal alcohol exposure has not yet been investigated. In the present study, phosphorylation of GSK3β in the hippocampus of rats prenatally exposed to alcohol was reduced by approx. 20% as compared to nonexposed controls (Fig 3A, *p* < 0.05). Interestingly, expression of ILK in the hippocampus was equivalent in both exposed and nonexposed animals (Fig 3B). Nonetheless, despite equivalent expression, changes in ILK activity may impair downstream phosphorylation. An ILK kinase assay revealed that ILK activity was indeed reduced by approximately 60% in the hippocampus of exposed animals, as compared to nonexposed controls (Fig 3C). These observations appear to confirm our hypothesis that decreased ILK activity reduces GSK3β phosphorylation, and that this reduced phosphorylation may be responsible for the memory and LTP impairments observed in the exposed animals.

### Moderate Prenatal alcohol exposure altered synaptic AMPAR expression

**Fig 4:**
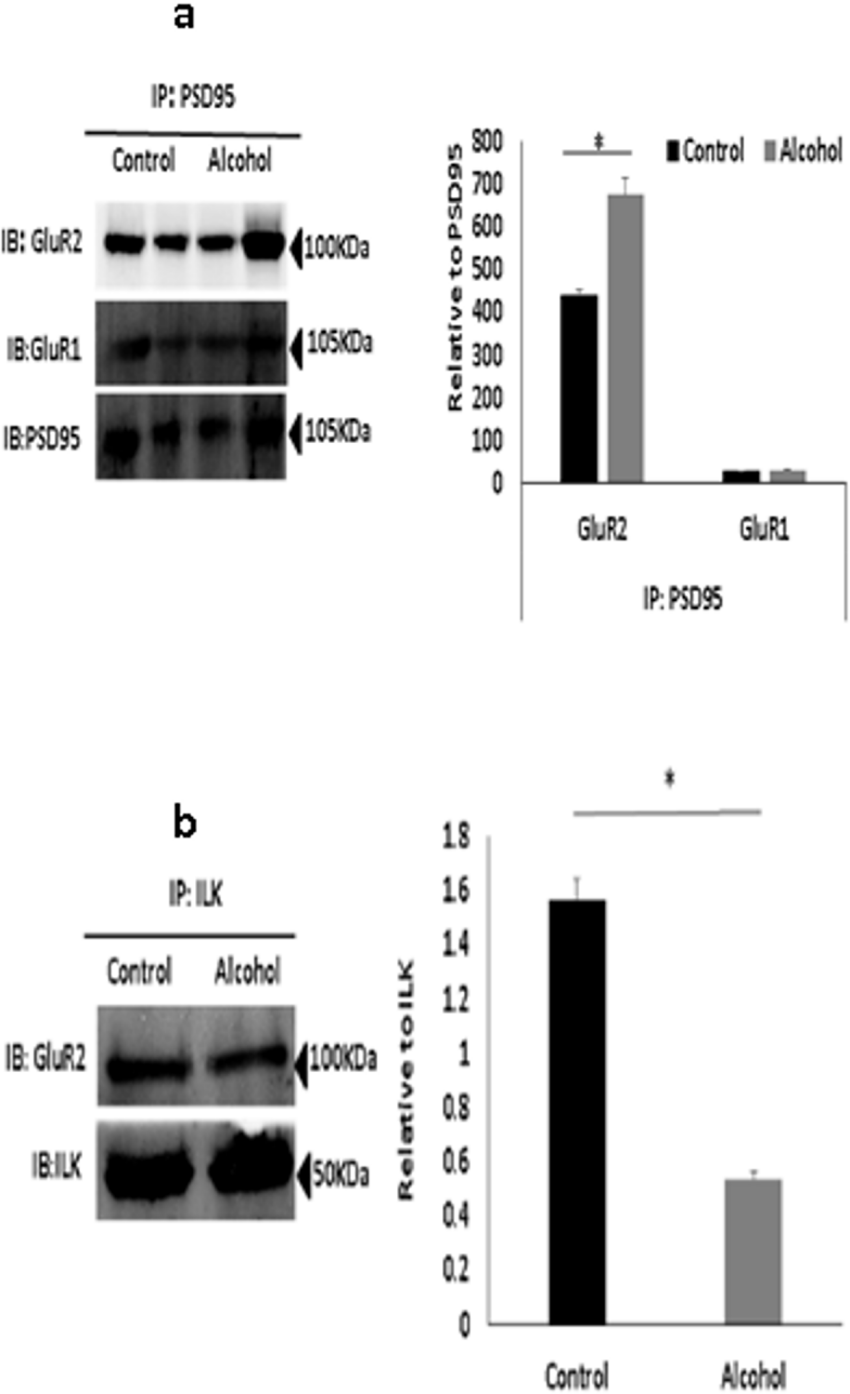
Prenatal alcohol may control GluR2 protein at the synapse through ILK. (a). Immunoprecipitation (IP) with anti–PSD-95 from pooled hippocampal protein lysates of rats prenatally-exposed to alcohol and nonexposed controls (*n* = 5). In exposed rats, precipitate of GluR2 increased as compared to controls, while precipitates of GluR1and ILK did not change. The same blotting membrane was reprobed with anti–PSD-95 as a control for PSD95 pull down. The quantitation is shown adjacent to the blot image (*p* < 0.05). (b) Immunoprecipitation (IP) with anti–ILK coprecipitates GluR2 from hippocampal protein lysates of exposed and nonexposed animals (*n* = 5). There was reduced interaction in the prenatal exposed rats as compared to the nonexposed controls. The western blot analysis of immunoprecipitated ILK with anti–ILK antibody was used to confirm equal ILK immunoprecipitation. The quantitation is shown adjacent to the blot image (*p* < 0.05).

ILK is known to interact with PSD95, an important scaffolding protein present at the synapse that interacts with several surface receptors. To understand the changes in AMPAR expression at the synaptic surface, we performed protein immunoprecipitation assays using anti-PSD95 antibody [37], and synaptic expression of GluR1, GluR2 and ILK was measured (Fig 4A). Synaptic expression of GluR2 was increased while synaptic expression of GluR1 was unchanged in animals exposed to alcohol prenatally. Increased GluR2 expression suggest an increase in calcium impermeable receptors at the synapse (GluR2 containing), which reduces the probability of action potential generation and, consequently, affect the NMDA-dependent LTP generation unlike calcium permeable (GluR2 lacking) receptors. Furthermore, calcium permeable receptors may also help in maintaining the availability of calcium ions necessary for LTP maintenance [38].

### Reduced ILK activity in prenatal alcohol exposure impaired ILK-AMPA receptor interaction

LTP induction requires GluR2-lacking inwardly rectifying AMPA receptor incorporation at synapse followed by replacement of GluR2-containing receptors (i.e., a GluR1 to GluR2 switch) [39]. Therefore, regulation of GluR2 receptors at the synapse is essential for LTP maintenance [40]. ILK interacts with surface glutamate receptors and proteins at the post-synaptic density, and it may act as a scaffolding protein by mediating phosphorylation and modulating trafficking of glutamate receptors to the surface. ILK immunoprecipitation assays used to assess GluR2 binding to ILK revealed a reduced interaction of GluR2 with ILK in animals exposed to alcohol in the prenatal period (Fig 4B). This impaired interaction suggests that ILK may play a crucial role in GluR2 stability at the synapse, resulting in reduced LTP.

### Moderate prenatal alcohol exposure resulted in reduced proBDNF/BDNF ratio

**Fig 5:**
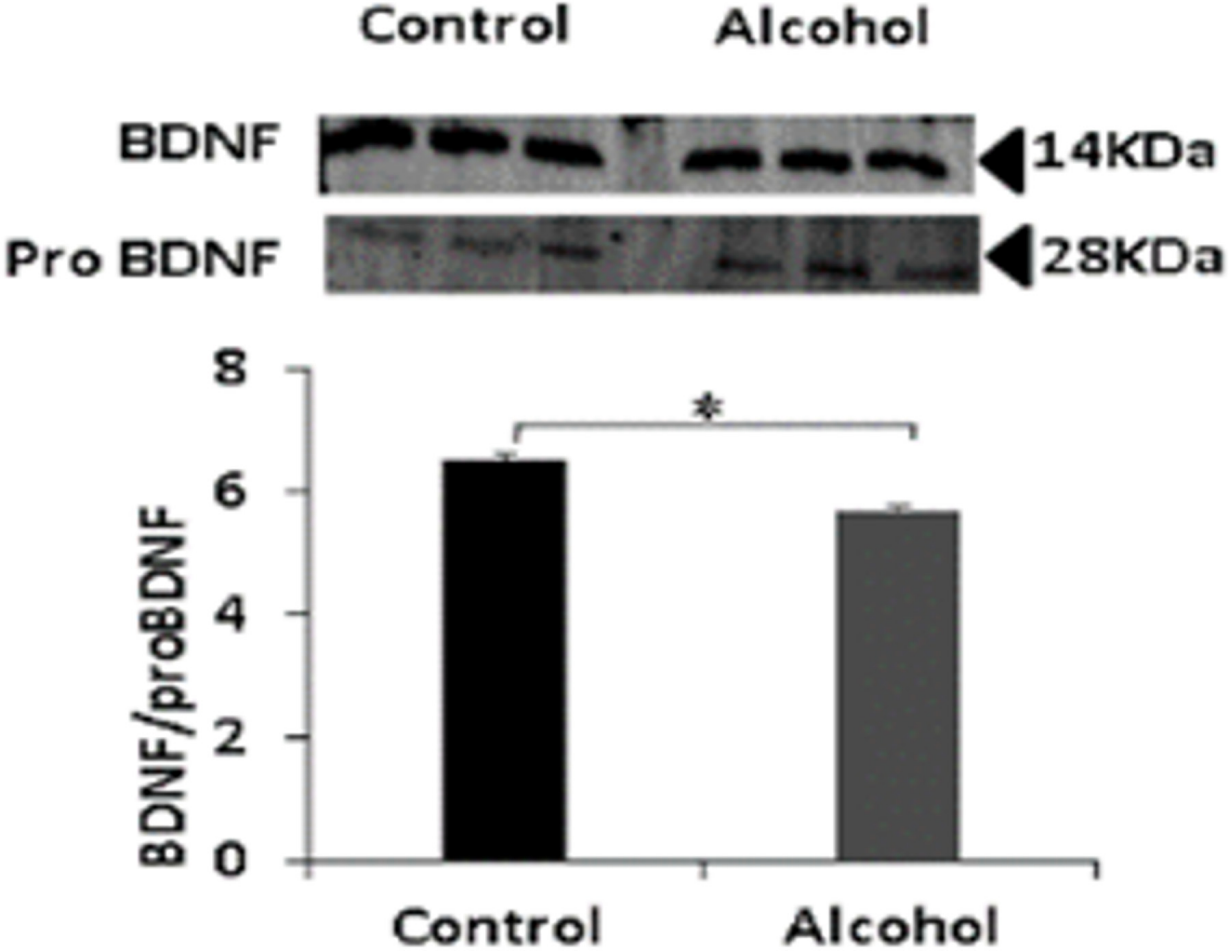
Prenatal alcohol exposure reduces mature BDNF expression. Western blot analysis of expression of mature BDNF and proBDNF proteins in hippocampal lysates from prenatal alcohol exposed and nonexposed rats (*n* = 4). Densitometry analysis shows that BDNF to proBDNF ratio decreased in prenatal alcohol exposed as compared to nonexposed rats (*p* < 0.05).

Expression of the mature form of BDNF is associated to increased plasticity and cell survival mechanisms in neurons [41]. Reduced mature BDNF can significantly impair ILK activity and downstream signaling processes [17, 41]. Contrarily, expression of proBDNF is associated to decreased neuronal survival and reduced synaptic plasticity [42]. Thus, the ratio of BDNF/proBDNF can provide a justification for reduced ILK activity at hippocampal glutamatergic synapses in the prenatal alcohol exposed rats. In our prenatal alcohol exposure model, we observed increased proBDNF and reduced BDNF; hence, the ratio of BDNF/proBDNF was significantly lower (p<0.05) than in nonexposed animals (Fig 5). This finding is consistent with earlier studies [43], and suggests that alcohol-induced reductions in the BDNF/proBDNF ratio contribute to the cognitive deficits observed in FASD animals.

## Discussion

Exposure to teratogens such as alcohol, nicotine, and cocaine during fetal development can modulate glutamate receptor and downstream signaling. Gestational exposure to alcohol results in reductions in brain size, with the cerebellum and corpus callosum being gravely affected by this exposure [44, 45]. Prenatal exposure to alcohol can also produce severe to moderate damage to hippocampal neurons [46]. The hippocampus is considered to be the ‘sorting region’ of the brain, categorizing inputs from other parts of the brain and beginning the process of storage of information into long- and short-term memory [47]. The Schaffer collateral pathway (CA3 to CA1) of the hippocampus plays an integral role in memory formation; therefore, any change in protein expression or interaction at the CA1 region could lead to memory impairments [48]. The hippocampus is essential for acquisition and performance of spatial and contextual memory tasks; thus, hippocampal function can be assessed behaviorally with procedures that require spatial or contextual information. In the present study, we used contextual fear conditioning to assess hippocampal memory in a rodent model of FASD, and observed marked deficits in short-term memory formation in exposed animals as compared to nonexposed controls. Behavioral assessment of hippocampal function was followed by field potential recording from the Schaffer collateral pathway. Consistent with the behavioral memory assessment, we observed LTP deficits that suggest that function of the hippocampus was dramatically affected by prenatal alcohol exposure.

AMPARs (fast excitatory glutamate receptors) play a crucial role in LTP induction and maintenance. AMPAR regulation at the synaptic surface controls NMDAR-dependent synaptic plasticity at the CA3-CA1 Schaffer collateral pathway [49, 50]. Some preliminary studies from our laboratory suggest that the interaction of ILK with glutamate receptors is of relevance in neurodegenerative diseases [15, 18] because changes in ILK expression can produce changes in downstream signaling molecules as well as changes in its scaffolding property. Interestingly, we observed no effects of prenatal alcohol exposure on ILK expression. However, there was reduced activity of ILK in exposed animals as compared to nonexposed controls. This suggests that ILK activity could be of high importance in synaptic plasticity after prenatal alcohol exposure. ILK is also present at the synaptic surface; therefore, changes in its activity may lead to changes in synaptic receptor phosphorylation. Surface AMPAR composition (measured by PSD95 immunoprecipitation assays) was altered by prenatal alcohol exposure, with increased expression of calcium-impermeable GluR2 receptors, and no change in expression of calcium-permeable GluR1 receptors. LTP maintenance requires a calcium permeable GluR1 to calcium impermeable GluR2 switch after LTP induction [51, 52]. Due to calcium impermeability, increased GluR2 containing receptors could impair LTP induction and maintenance, as appears to be the case in our exposed animals. Furthermore, we observed reduced GluR2 interaction with ILK in the hippocampus. Such reduced interaction may influence GluR2 stabilization through reduced phosphorylation of target amino acid residues because such reduced phosphorylation may help stabilize GluR2 receptors on the synaptic surface. A potential target residue is Ser^880^ on GluR2 [53]. Thus, the present results identify ILK as an important molecule, whose activity warrants further research in models of FASD-induced cognitive deficits.

## Conclusion

**Fig 6:**
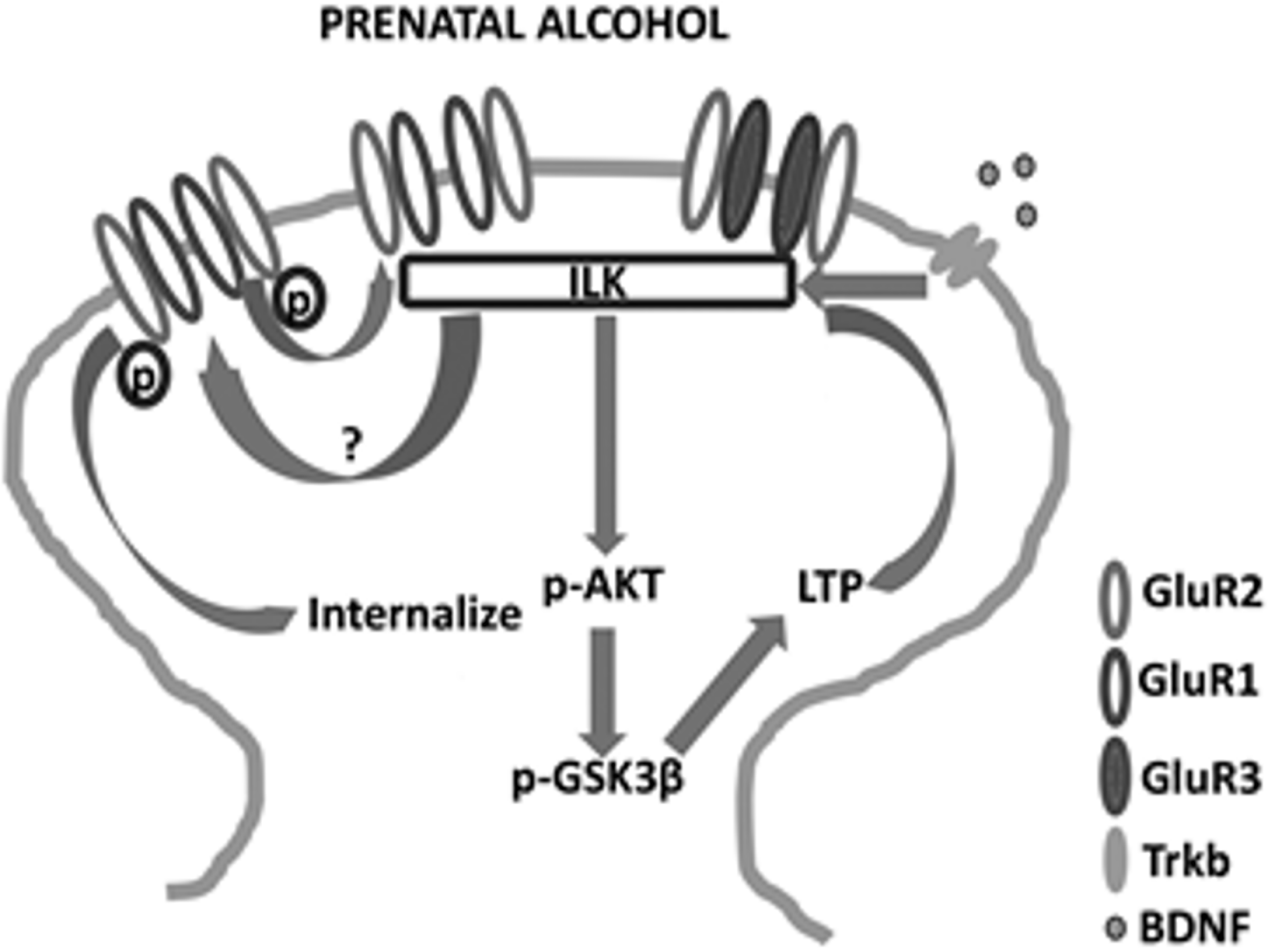
Schematic diagram showing the probable mechanism through which prenatal alcohol modulates ILK and affects plasticity. ILK may affect an unknown amino acid phosphorylation which may help in GluR2 receptor internalization. Reduced ILK activity may reduce the target phosphorylation and stabilize GluR2 at the surface thereby increase GluR2 at the synapse. Increased GluR2 and downstream active GSK3β may reduce LTP induction and maintenance. The probable cause of reduced ILK activity could be less mature BDNF availability in the brain of animals prenatally exposed to alcohol.

The present study constitutes the first report highlighting the involvement of ILK in FASD-related memory impairments and synaptic plasticity. From this study, we conclude a close association of impaired ILK pathway and synaptic plasticity deficits in prenatal alcohol exposed rat model. Reduced ILK activity could be due to reduced BDNF to proBDNF ratio. The reduced kinase activity and diminished interaction to GluR2 AMPAR could be responsible for increased stabilization of GluR2 containing receptors at the synapse. The increased calcium impermeable AMPAR is responsible for impaired LTP induction and maintenance. Reduced LTP can also be due to increased GSK3β activation which could affect receptor trafficking and protein expression required for LTP maintenance. These findings from this research work have been summarized in Fig 6. Furthermore, our study suggests that FASD-related memory impairments can be due to impaired ILK pathway.
